# Identification of hydrogen peroxide production-related genes in *Streptococcus sanguinis* and their functional relationship with pyruvate oxidase

**DOI:** 10.1099/mic.0.039669-0

**Published:** 2011-01

**Authors:** Lei Chen, Xiuchun Ge, Yuetan Dou, Xiaojing Wang, Jenishkumar R. Patel, Ping Xu

**Affiliations:** The Philips Institute of Oral and Craniofacial Molecular Biology, Virginia Commonwealth University, Richmond, VA 23298-0566, USA

## Abstract

Hydrogen peroxide (H_2_O_2_), an important substance produced by many members of the genus *Streptococcus*, plays important roles in virulence and antagonism within a microbial community such as oral biofilms. The *spxB* gene, which encodes pyruvate oxidase, is involved in H_2_O_2_ production in many streptococcal species. However, knowledge about its regulation and relation with other genes putatively involved in the same pathway is limited. In this study, three genes – *ackA, spxR* and *tpk* – were identified as contributing to H_2_O_2_ production in *Streptococcus sanguinis* by screening mutants for opaque colony appearance. Mutations in all three genes resulted in significant decreases in H_2_O_2_ production, with 16–31 % of that of the wild-type. H_2_O_2_ production was restored in the complemented strains. Antagonism against *Streptococcus mutans* by these three *S. sanguinis* mutants was reduced, both on plates and in liquid cultures, indicating the critical roles of these three genes for conferring the competitive advantage of *S. sanguinis.* Analysis by qPCR indicated that the expression of *spxB* was decreased in the *ackA* and *spxR* mutants and significantly increased in the *tpk* mutant.

## INTRODUCTION

Hydrogen peroxide (H_2_O_2_) is produced by many members of the genus *Streptococcus* (García-Mendoza *et al.*, 1993; Kreth *et al.*, 2005; Ramos-Montañez *et al.*, 2008) and is important in three aspects. First, H_2_O_2_ is reported to correlate with virulence in *Streptococcus pneumoniae* (Auzat *et al.*, 1999; Ramos-Montañez *et al.*, 2008; Weiser *et al.*, 1994). *S. pneumoniae* undergoes spontaneous phase variation resulting in opaque and transparent colony forms, and the differences in colony opacity correlate with virulence (Weiser *et al.*, 1994). Recent research indicates that transparent variants are more proficient in colonization, with more production of teichoic acid and H_2_O_2_, but with less production of capsule than opaque variants (Ramos-Montañez *et al.*, 2008). Studies also suggest that the H_2_O_2_ produced by *Streptococcus pyogenes* acts as a potential virulence factor by exerting direct damage to host tissues (Ginsburg & Sadovnic, 1998; Ginsburg & Varani, 1993). Second, H_2_O_2_ production is related to competition and co-existence within microbial communities such as oral biofilms. Many streptococci are able to produce inhibitory substances such as H_2_O_2_ to reduce the growth of co-resident micro-organisms. For example, *S. sanguinis* can produce H_2_O_2_ that will inhibit growth of *Staphylococcus aureus* (Uehara *et al.*, 2006). *S. sanguinis* and *Streptococcus gordonii* demonstrate antagonistic activity against *Streptococcus mutans* via H_2_O_2_ production (Kreth *et al.*, 2005). Other studies also investigated the inhibitory capacity of H_2_O_2_ produced by various species of oral streptococci (García-Mendoza *et al.*, 1993; Kreth *et al.*, 2005, 2008). Recently, H_2_O_2_ was also shown to contribute to the release of DNA from *S. sanguinis* and *S. gordonii*, which appears to support oral biofilm formation and facilitate exchange of genetic material among competent strains (Kreth *et al.*, 2009). Third, H_2_O_2_ is a by-product of aerobic metabolism (Jakubovics *et al.*, 2002). Many streptococci produce relatively large amounts of H_2_O_2_ during aerobic growth by the action of oxidase enzymes such as pyruvate oxidase and NADH oxidase (Auzat *et al.*, 1999; Tittmann *et al.*, 2005).

*S. sanguinis* is a member of the human indigenous oral microflora and one of the major microbes colonizing teeth (Kuramitsu *et al.*, 2007; Rosan & Lamont, 2000). It is also one of the most common causative agents of infective endocarditis (Douglas *et al.*, 1993; Mylonakis & Calderwood, 2001; Tleyjeh *et al.*, 2005). On the other hand, *S. sanguinis* is considered an antagonistic bacterium against *S. mutans* (Becker *et al.*, 2002; Caufield *et al.*, 2000). Relatively high proportions of *S. sanguinis* are generally found in dental plaque with lower levels of *S. mutans.* High levels of *S. mutans* in the oral cavity correlate with low levels of *S. sanguinis* (Caufield *et al.*, 2000; Ge *et al.*, 2008b).

In *S. pneumoniae*, the function of pyruvate oxidase (SpxB) in H_2_O_2_ production has been well characterized (Weiser *et al.*, 1994). Pyruvate oxidase plays several critical roles in pneumococcal metabolism and virulence (Ramos-Montañez *et al.*, 2008), including phase variation (Overweg *et al.*, 2000; Pericone *et al.*, 2000, 2002). Though the genetic mechanism behind the phase variation has remained unclear, this morphological change has been successfully used in *S. pneumoniae* to identify SpxR, a regulator of *spxB* required for *spxB* transcription and for full virulence in a murine model of infection (Ramos-Montañez *et al.*, 2008).

Although H_2_O_2_ production is involved in important metabolic pathways, potential pathogenic virulence, and interspecies competition, knowledge of its metabolic basis and regulation remains limited in *S. sanguinis*. The *spxB* gene is involved in H_2_O_2_ production (Weiser *et al.*, 1994; Ramos-Montañez *et al.*, 2008), but its regulation and relation with other genes putatively involved in the same pathway are still unclear. SpxB is a decarboxylase that catalyses the conversion of pyruvate, inorganic phosphate (Pi) and molecular oxygen (O_2_) to H_2_O_2_, carbon dioxide (CO_2_) and acetyl phosphate, which acts as a high-energy phosphoryl group donor (Ramos-Montañez *et al.*, 2008):
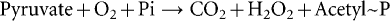
During a study of gene deletion mutants of *S. sanguinis* SK36, we noticed that some mutants showed opaque colonies, suggesting a possible deficiency in H_2_O_2_ production. To identify novel genes relevant to H_2_O_2_ production and their relationship with *spxB*, we screened the single-gene mutant library being constructed in our lab to find H_2_O_2_ production-defective mutants. Here, we describe three genes that are involved in pyruvate oxidase-related H_2_O_2_ production and antagonism against *S. mutans*, together with their functional relationship with pyruvate oxidase in *S. sanguinis*.

## METHODS

### Bacterial strains and growth media.

The strains used are described in Table 1. *S. sanguinis* strain SK36 (obtained from Dr Mogens Kilian, Århus University, Denmark) was isolated from human dental plaque (Kilian & Holmgren, 1981). This strain and its derivatives were routinely grown in brain heart infusion broth (BHI; Difco) supplemented with 1.5 % (w/v) agar under microaerobic conditions (7.2 % H_2_, 7.2 % CO_2_, 79.6 % N_2_ and 6 % O_2_) in an Anoxomat jar (Spiral Biotech) at 37 °C as described previously (Ge *et al.*, 2008a; Paik *et al.*, 2005). *S. mutans* UA159 or its derivative was also routinely grown in BHI under microaerobic conditions as for *S. sanguinis*. When needed, medium was supplemented with kanamycin (500 μg ml^−1^), chloramphenicol (5 μg ml^−1^) or erythromycin (10 μg ml^−1^). The mutants were named by using “*ssx*” to refer the corresponding *ssa* gene in the NCBI database.

### Mutant construction and complementation.

For the construction of precise single gene deletion mutants in *S. sanguinis* SK36, we developed a PCR-based recombinant method employing linear DNA for deletion construction *in vitro* (P. Xu and others, unpublished data). Briefly, for each targeted gene, three sets of primers were designed to amplify a linear DNA fragment containing the kanamycin resistance cassette (*aphA-3*) (Turner *et al.*, 2009) with two flanking arms of DNA upstream and downstream of the targeted gene. The linear recombinant PCR amplicon was directly transformed into *S. sanguinis* competent cells as described previously (Ge *et al.*, 2008a). A 96-well high-throughput format was used to generate a genome-wide mutant library. The mutants were confirmed by PCR and RT-PCR analyses.

To construct the complemented strain, a DNA fragment containing the targeted gene followed by a selectable marker (either chloramphenicol or erythromycin resistance cassette) (Turner *et al.*, 2009) was integrated via double homologous recombination into the corresponding mutant (Table 1) to replace the kanamycin resistance cassette. Chloramphenicol- or erythromycin-resistant and kanamycin-sensitive transformants were selected and confirmed by PCR analysis.

### Screening for H_2_O_2_ production-defective mutants by opaque colony morphology.

For opaque colony observation, 5 μl overnight culture of *S. sanguinis* SK36 or different mutants was spotted on the surface of BHI plates and incubated at 37 °C under microaerobic conditions. For catalase-containing plates, 100 μl catalase from bovine liver (Sigma) was spread on the BHI plate surface (∼880 U cm^−2^). The plates were air-dried for 10 min in a hood before bacterial inoculation. Bacterial suspension (5 μl) was spotted on the catalase-containing plate and incubated at 37 °C under microaerobic conditions for 2 days. The opaque colonies on the plate were recorded and photographed using a BioDoc-It imaging system. After obtaining the opaque mutants in the assays, the integrity of the mutations was confirmed by using PCR amplification and sequencing.

### H_2_O_2_ release assays.

H_2_O_2_ production was quantified using the Amplex Red hydrogen peroxide/peroxidase assay kit (Invitrogen) as described by the manufacturer, with minor modifications (Ramos-Montañez *et al.*, 2008). Briefly, 100 μl reaction mixture (50 mM Amplex Red reagent, 0.1 U horseradish peroxidase ml^−1^ in 0.05 M sodium phosphate buffer, pH 7.4) was dispensed into wells of a 96-well microtitre plate and warmed to 37 °C for 10 min. Exponential cultures of *S. sanguinis* strains were grown in BHI to OD_450_ 0.15–0.2, centrifuged and then resuspended in fresh BHI to OD_450_ approximately 0.06. An aliquot of freshly resuspended cells (20 μl) was added in triplicate to the 100 μl pre-warmed reaction mixture. A series of H_2_O_2_ concentration standards diluted in BHI together with BHI as the blank control were also included in the plate. The microtitre plate was incubated at 37 °C in a FLUOstar plate reader under aerobic conditions and absorbance was read at 563 nm every 5 min for 20 min. Rates of H_2_O_2_ production were calculated and normalized to the OD_450_ of the cell suspensions. Final values are shown relative to that of the wild-type strain, SK36. Paired *t*-test was used for statistical analysis.

### Competition assays on plates.

To determine the inhibitory effect of *S. sanguinis* against *S. mutans*, a previously described protocol (Kreth *et al.*, 2005, 2008) was employed with the following modifications. Briefly, 5 μl of an overnight culture of *S. sanguinis* SK36 or its derivative in BHI medium was inoculated onto a BHI agar plate. After incubation overnight (16 h), 5 μl of *S. mutans* UA159 was inoculated next to an *S. sanguinis* colony. Colonies were inoculated so that they were just touching each other. The plate was incubated again for another night. Growth inhibition was evaluated based on the distance of the inhibition zone from the edges of both colonies.

### Competition assays in liquid media.

This was performed as described by Kreth *et al.* (2008). Cells of *S. sanguinis* mutants and *S. mutans smx_42*, a chloramphenicol-resistant derivative of *S. mutans*, were grown in BHI medium overnight and adjusted to the same OD_660_ value. *S. sanguinis* or its mutants (3 μl of each) and *S. mutans smx_42* (3 μl) were mixed with 200 μl fresh BHI medium in 96-well microtitre plates in triplicate. The cells were incubated overnight in static culture under microaerobic conditions. Cells were dispersed by vigorous pipetting and serial dilutions were plated on BHI agar plates supplemented with chloramphenicol in triplicate and the c.f.u. was determined.

### RNA extraction and qPCR analyses.

Total RNA was prepared from the cells growing in late exponential phase in BHI medium under microaerobic conditions to OD_450_ 0.6–1.0. Cells were lysed after lysozyme treatment and mechanical disruption using FastPrep lysing matrix B (Qbiogene). RNA was isolated by using the RNeasy mini kit (Qiagen). DNA was removed from the RNeasy mini kit column by DNase I treatment. Total RNA was quantified using a NanoDrop ND 1000 spectrophotometer. First-strand cDNA synthesis was performed in a 20 μl reaction mixture containing 100 ng RNA, 0.5 μl random primers (3 μg μl^−1^), 1.0 μl dNTP mix (10 mM each dNTP), 1.0 μl 100 mM DTT, 1.0 μl RNAout (40 U; Invitrogen) and 0.5 μl SuperScript III reverse transcriptase (200 U μl^−1^) in first-strand buffer (Invitrogen). Reactions lacking reverse transcriptase were prepared in parallel as controls for possible DNA contamination. First strand cDNA from each reaction was subjected to 80-fold dilutions, and 2 μl of each dilution was used as template for each PCR. Quantitative real-time PCR was performed in reactions containing 5 μl SYBR Green PCR master mix (Applied Biosystems), 1 μl each PCR primer (2 mM) using the ABI 7500 fast real-time PCR system. The housekeeping gene *gyrA* was used as a normalization control. The data were collected and statistically analysed from triplicates. Serial dilutions of chromosomal DNA from wild-type strain SK36 were used for standard curves.

## RESULTS

### H_2_O_2_ production determines colony morphology in *S. sanguinis* SK36

During the process of creating a genome-wide single gene deletion mutant library of *S. sanguinis* SK36, it was found that some mutants showed different colony morphologies. In comparison with the semi-transparent colony of the wild-type strain SK36, certain mutants presented an opaque colony when grown on BHI agar plates under microaerobic conditions. It was reported previously that the colony morphology variation between transparent and opaque colonies in *S. pneumoniae* is related to H_2_O_2_ production and can be detected on tryptic soy agar plates by the addition of catalase (Weiser *et al.*, 1994). Such an opaque colony marker has been used to identify genes involved in H_2_O_2_ production in *S. pneumoniae* (Ramos-Montañez *et al.*, 2008). To investigate the existence of similar morphological variation in *S. sanguinis* and to establish a condition to screen genes involved in H_2_O_2_ production, *S. sanguinis* SK36 was cultured on BHI plates with and without the addition of excess catalase to hydrolyse peroxides. The colony opacity was compared to examine the effect of catalase. We found that SK36 colonies changed from semi-transparent to opaque after incubation for 2 days when catalase was added (data not shown). This showed that in *S. sanguinis*, the opaque colony variations were related to H_2_O_2_ production. The result also suggested that H_2_O_2_ production-defective mutants of *S. sanguinis* SK36 might be identified by colony morphology in our system. We hypothesized that the mutants with opaque colonies had lower H_2_O_2_ production.

Next, to identify the potential genes involved in H_2_O_2_ production, we screened over 1000 available single gene deletion mutants for variation in colony morphology to identify potential H_2_O_2_-production-defective mutants, as described above. Four mutants showing obvious opaque colonies were identified. The morphological variations of the four mutants were further confirmed by comparison with the wild-type strain SK36 on BHI plates, including a control strain *ssx_0169* with kanamycin resistance (data not shown) to determine that the kanamycin resistance gene did not interfere with the phenotypes being investigated. This control strain was selected because it was demonstrated that the *ssa_0169* gene did not affect important cellular phenotypes (Turner *et al.*, 2009). The deletion locus of each mutant was confirmed to have the expected structure by PCR analysis and DNA sequencing. One of the mutants (*ssx_0391*) had a deletion in the *spxB* gene, whose product, pyruvate oxidase, is known to catalyse the production of H_2_O_2_ (Ramos-Montañez *et al.*, 2008). The other three genes identified were *ackA*, encoding acetate kinase, *spxR*, encoding a conserved hypothetical protein, and *tpk*, encoding thiamine pyrophosphokinase (Table 1). The four opaque mutants (including *ssx_0391*) were further characterized.

### Opaque mutants have reduced rates of H_2_O_2_ production

**Fig. 1.**
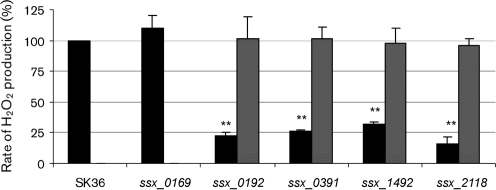
H_2_O_2_ production in *S. sanguinis* strains. H_2_O_2_ production normalized to culture densities was determined relative to that produced by the wild-type strain SK36. Data indicate mean±sd from three biological repeats. Statistical significance is indicated (**P<0.01). Black bars, SK36 or a mutant; grey bars, complemented strain.

Since H_2_O_2_ production is proposed to relate to the opaque morphology, we next quantified H_2_O_2_ production in the four mutants identified above, and compared it with that of the wild-type strain SK36. The control strain *ssx_0169* was also included in this analysis. All four opaque mutants displayed significantly reduced rates of H_2_O_2_ production compared with the semi-transparent parent strain SK36 (Fig. 1). H_2_O_2_ production rates of the mutants were only 16–31 % of that of SK36. Similar H_2_O_2_ production to the wild-type strain SK36 was found in the kanamycin-resistant control strain (*ssx_0169*). Though each of the four opaque mutants displayed decreased rates of H_2_O_2_ production, none of them lost the capacity of H_2_O_2_ production completely, including the *spxB* mutant. It should be noted that the intact ORF of each mutant was precisely deleted in each of our mutants, so it was impossible that any partial gene function remained. This suggested that the pyruvate oxidase activity might not be the only oxidase activity responsible for H_2_O_2_ production.

Next, to ensure that these identified genes function in H_2_O_2_ production, we checked the H_2_O_2_ production in the relative single gene mutants of their upstream and downstream genes (i.e. *ssx_0190*, *ssx_0191*, *ssx_0193* and *ssx_0195*; *ssx_1494*, *ssx_1493*, *ssx_1490* and *ssx_1489*; *ssx_2120*, *ssx_2119*, *ssx_2117* and *ssx_2116.* The mutant for *ssa_1491* is not available because the gene was found to be essential). There was no statistically significant defect in H_2_O_2_ production by any of these mutants compared with that of the wild-type. This result supports the hypothesis that the defects in H_2_O_2_ production in the identified mutants are not related to the neighbouring genes. To ensure this, we introduced the genes back to the mutants. A chloramphenicol resistance cassette was placed downstream of each gene for selection. After obtaining the complemented strains, their morphology and H_2_O_2_ production were examined. The results showed that the morphology of three strains, *ssx_0391C*, *ssx_1492C* and *ssx_2118C* (Table 1), was restored to semi-transparent and the rates of H_2_O_2_ production were restored to the wild-type level (Fig. 1). In the first attempt to complement *ssx_0192* gene function, we failed to fully restore the phenotype. We then examined the mRNA level of downstream genes *ssa_0193*, *ssa_0195* and *ssa_0197* in the mutant *ssx_0192*, which did not show significant changes compared with that of the wild-type. Given these data, we deduced that there might be some errors with the complemented strain. We therefore performed this complementation again employing an erythromycin resistance cassette (*pSerm*). The resulting strain *ssx_0192C* was successfully restored for H_2_O_2_ production (Fig. 1). All of the data indicated that the identified genes are involved in H_2_O_2_ production.

### Opaque mutants demonstrate reduced antagonistic activity against *S. mutans* UA159 both on plates and in liquid media

**Fig. 2.**
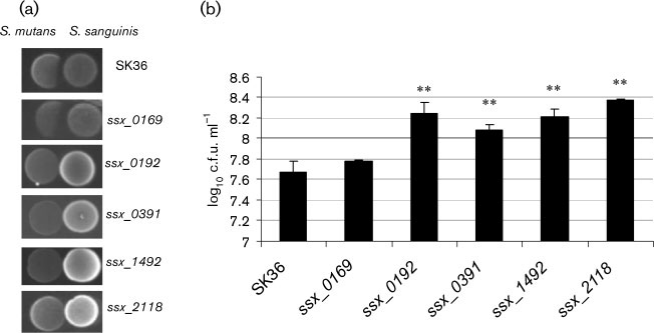
Inhibitory effect of *S. sanguinis* on *S. mutans*. (a) Inhibition assay on plates. Overnight cultures of different *S. sanguinis* strains were inoculated on BHI plates, which were incubated for 16 h at 37 °C under microaerobic conditions. *S. mutans* UA159 was then inoculated next to the pioneer colonizer, and the plates were further incubated overnight and photographed. (b) Inhibition assay in liquid media. Overnight cultures of *S. sanguinis* SK36 or mutants were adjusted to the same optical density and mixed with the *S. mutans* UA159 (Cm) in fresh BHI medium. After overnight growth, the cells were serially diluted and plated on BHI plates supplemented with chloramphenicol. The log_10_ c.f.u. ml^−1^ values±sd of *S. mutans* UA159 are shown (data are from triplicate experiments) (**P<0.01 relative to the values obtained for the SK36 mixture).

Because the formation of H_2_O_2_ in *S. sanguinis* plays an important role in interspecies interactions within the oral microflora, we performed competition assays to examine whether the four H_2_O_2_-defective mutants showed any difference from the parent strain, SK36, in their capacity for antagonism against a primary dental cariogen, *S. mutans*. We first examined the antagonistic activity on agar plates. *S. sanguinis* and *S. mutans* cells were spotted next to one another on agar plates. The inhibition zones of *S. sanguinis* and the mutants against *S. mutans* were determined (Fig. 2a). The results showed that all four mutants lost the ability to inhibit *S. mutans* UA159 on BHI plates under microaerobic conditions (Fig. 2a).

To further quantify the antagonism of the H_2_O_2_-defective mutants against *S. mutans*, we performed competition assays in liquid culture using mixed species. We first constructed a chloramphenicol-resistant control strain of *S. mutans* by integrating the chloramphenicol resistance gene *magellan2* (Turner *et al.*, 2009) into the *S. mutans* UA159 chromosome. The *smu.42* gene encoding a hypothetical protein (SMU.42) which did not affect its sensitivity to antagonism by *S. sanguinis* (data not shown) was selected as the target location for integration in the *S. mutans* genome. Because the *S. mutans* derivative can be distinguished from *S. sanguinis* on chloramphenicol selection agar plates, the inhibition effect of *S. sanguinis* on *S. mutans* could be determined by bacterial colony numbers on agar plates supplemented with chloramphenicol. We mixed the same amount of each *S. sanguinis* mutant, as assessed by OD_660_, with *S. mutans* and co-cultured the two species mixture. *S. mutans* cells were counted on BHI plates supplemented with chloramphenicol after 48 h. This indicated that all four mutants were less able to inhibit *S. mutans* in liquid culture compared with the wild-type strain, SK36, and the control strain *ssx_0169* (Fig. 2b).

### Transcriptional level of *spxB* in H_2_O_2_ production-defective mutants

**Fig. 3.**
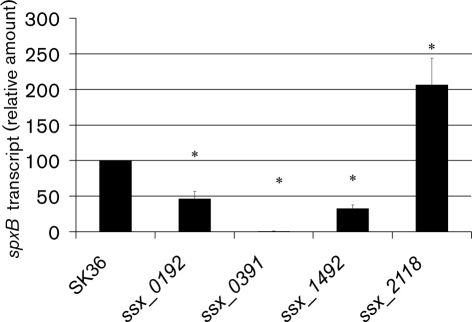
*spxB* transcription in *S. sanguinis* SK36 and mutants. RNA preparation and qPCR were performed as described in Methods. The amount of *spxB* transcript was normalized to that of *gyrA*. Data shown are mean±sd from three biological replicates. *Significant difference compared with SK36, *P*<0.05.

To examine whether *spxB* expression changes in the mutants with decreased H_2_O_2_ production, we determined the transcriptional level of *spxB* by real-time qPCR (Fig. 3). The results showed that the expression of *spxB* in *ssx_0192* and *ssx_1492* decreased significantly compared with SK36. The significant decrease in *spxB* transcription suggested that the effects of the deleted gene products in *ssx_0192* and *ssx_1492* on H_2_O_2_ production might occur via SpxB. In contrast, the *ssx_2118* mutant demonstrated increased expression of *spxB*, indicating that SSA_2118 affects H_2_O_2_ production by a mechanism other than affecting *spxB* expression (Fig. 3).

## DISCUSSION

In this study, we describe three genes involved in the production of H_2_O_2_ and the preliminary study of their effects on *spxB* expression in *S. sanguinis*. We also show that all the genes involved in the production of H_2_O_2_ identified here were also critical for the antagonism of *S. sanguinis* against *S. mutans*.

The three non-*spxB* mutants identified in this study demonstrated defects in H_2_O_2_ production similar to that in the *spxB* mutant. SSA_0192 is annotated as acetate kinase (Xu *et al.*, 2007), which converts acetyl phosphate, the other product derived from the decarboxylation of pyruvate besides CO_2_ and H_2_O_2_, to acetate. Our results indicated that *spxB* expression was reduced in the deletion mutant *ssx_0192*. A possible mechanism of this regulation might be that the gene deletion in *ssx_0192* caused acetyl phosphate accumulation, which caused feedback suppression of *spxB* expression (Wang *et al.*, 1999). We tried to determine the acetyl phosphate concentration using the hydroxamate assay (Gorrell *et al.*, 2005) in *ssx_0192* and the wild-type strain to examine this hypothesis. However, the acetyl phosphate concentrations in both strains were too low to give a reliable result. The gene product in *ssx_1492* (SSA_1492) showed high identity (76 % identity in amino acid sequence) to SpxR in *S. pneumoniae*, which was identified as a regulator of *spxB* (Ramos-Montañez *et al.*, 2008). The conserved domain analysis of SSA_1492 showed that the protein contains a putative helix–turn–helix domain (Ramos-Montañez *et al.*, 2008) located at the amino terminus, followed by a DRTGG-CBS domain, which are hypothesized to bind to DNA and adenosyl compounds (such as AMP and ATP), respectively. Combined with the significantly decreased expression of *spxB* observed in the *ssx_1492* mutant, our data suggest that SSA_1492 might act as a positive regulator of *spxB* (Kemp, 2004; Ramos-Montañez *et al.*, 2008; Rigali *et al.*, 2002; Scott *et al.*, 2004). It was hypothesized that SpxR in *S. pneumoniae* regulates *spxB* transcription in response to energy and metabolic state, and the SpxR regulon includes comparatively few genes (Ramos-Montañez *et al.*, 2008). As some other species of streptococci, including *S. mutans*, *S. pyogenes* and *Streptococcus agalactiae* (group B Streptococcus), lack *spxB* but contain homologues of SpxR (Ramos-Montañez *et al.*, 2008), presumably the regulatory targets of the SpxR homologues are species-specific. Our study suggests that the role of SpxR in regulating *spxB* is not confined to *S. pneumoniae*, because it seems to have the same function in *S. sanguinis*. The thiamine pyrophosphokinase encoded by *ssa_2118* catalyses the transfer of a pyrophosphate moiety from ATP to thiamine and produces thiamine pyrophosphate. Thiamine pyrophosphate has been reported to be an important cofactor for pyruvate oxidase activity (Carlsson & Kujala, 1984; Muller *et al.*, 1994; Tittmann *et al.*, 1998, 2005). The deletion of *ssa_2118*, therefore, presumably decreases the rate of H_2_O_2_ formation by decreasing the activity of pyruvate oxidase (Fig. 1). Our results indicated that SSA_2118 was required for H_2_O_2_ production. This is also consistent with the finding that a site-specific mutation of an amino acid in SpxB that is required for thiamine pyrophosphate binding reduces H_2_O_2_ production significantly in *S. pneumoniae* (Ramos-Montañez *et al.*, 2008). At the same time, it is possible that SSA_2118 involves H_2_O_2_ production by affecting not only SpxB but also other enzymes that require thiamine pyrophosphate. It was interesting that *spxB* expression in mutant *ssx_2118* showed a significant increase. All three non-*spxB* mutants exhibited decreased H_2_O_2_ production but the expression levels of *spxB* were distinct. We hypothesize that SSA_0192 and SSA_1492 were required for the normal expression of *spxB*, while SSA_2118 was required for the thiamine pyrophosphate, which is the cofactor for SpxB. Both SpxB and SSA_2118 were necessary for H_2_O_2_ production.

Our study suggests that it is practical to identify H_2_O_2_ production-defective mutants in *S. sanguinis* by their colony morphology variation. In *S. pneumoniae*, the proposed roles of *spxB* function and regulation in pneumococcal phase variation have been somewhat contradictory (Ramos-Montañez *et al.*, 2008). Some research suggested that *spxB* expression level was unlikely to directly determine colony morphology (Overweg *et al.*, 2000) since the *spxB* mutant still varied in colony morphology, while another study indicated that some opaque variants were later found to be defective in SpxB function (Pericone *et al.*, 2002). It has been suggested recently that SpxB does determine colony morphology and might play a role in phase variation (Belanger *et al.*, 2004). The contradiction may be related to other components contributing to the phenotype, such as a capsule. In *S. sanguinis*, our results indicated that it was not the expression of *spxB* that is responsible for the morphological variation, but the H_2_O_2_ the strain produces, because all of the four H_2_O_2_ production-defective mutants had an opaque appearance, and this appearance was not dependent on the expression of *spxB*. For example, the expression of *spxB* in mutant *ssx_2118* was significantly increased compared with that in the wild-type strain SK36 (Fig. 3), but the mutant *ssx_2118* still presented an opaque morphology, which is presumably due to the H_2_O_2_ production deficiency of the mutant.

In *S. pneumoniae*, a similar screening study was performed by Ramos-Montañez *et al.* (2008). From screening ∼232 000 colonies, seven spontaneous mutants were identified that showed opaque appearance; six of them were found to produce less H_2_O_2_ than the wild-type strain. All were related to *spxB* and one of the genes, *spxR*, was found to regulate *spxB* expression. In our study, in addition to *spxB* and *spxR*, we identified two other mutants, *ssx_0192* and *ssx_2118*, that had an opaque appearance and produced less H_2_O_2_. It is interesting that these genes were not identified in the *S. pneumoniae* study, even though we would expect that identical mutants in *S. pneumoniae* would have the same phenotype. This could be because their spontaneous screen carried out by Ramos-Montañez *et al.* (2008) was not saturating. It would be interesting to determine whether mutations in the *ssa_0192* and *ssa_2118* orthologues in *S. pneumoniae* (*spd_1853* and *spd_1779*) would have also demonstrated this phenotype. If so, they will be potential virulence factors in *S. pneumoniae*.

The competition between pioneer colonizing oral streptococci in the oral community is of continued interest. Our studies show that the four genes identified are critical for conferring a competition advantage to *S. sanguinis*. This might contribute to a better understanding of interspecies interactions within oral microbial communities and serve as a foundation on which the molecular mechanisms of H_2_O_2_ production and its regulation by oral streptococci could be elucidated.

## Figures and Tables

**Table 1.** Bacterial strains used in this study Cm, chloramphenicol; Em, erythromycin; Km, kanamycin.

**Strain**	**Phenotype or description**	**Source**
***S. sanguinis***		
SK36	Human plaque isolate	Kilian & Holmgren (1981)
*ssx_0169*	Km^r^; Δ*0169* : : *aphA-3*	This study
*ssx_0192*	Km^r^; Δ*ackA* : : *aphA-3*	This study
*ssx_0192*C	Em^r^; *ackA+* : : *pSerm*	This study
*ssx_0190*	Km^r^; Δ*ssa_0190* : : *aphA-3*	This study
*ssx_0191*	Km^r^; Δ*ssa_0191* : : *aphA-3*	This study
*ssx_0193*	Km^r^; Δ*ssa_0193* : : *aphA-3*	This study
*ssx_0195*	Km^r^; Δ*ssa_0195* : : *aphA-3*	This study
*ssx_0391*	Km^r^; Δ*spxB* : : *aphA-3*	This study
*ssx_0391*C	Cm^r^; *spxB+* : : *magellan2*	This study
*ssx_1492*	Km^r^; Δ*spxR* : : *aphA-3*	This study
*ssx_1492*C	Cm^r^; *spxR+* : : *magellan2*	This study
*ssx_1494*	Km^r^; Δ*ssa_1494* : : *aphA-3*	This study
*ssx_1493*	Km^r^; Δ*ssa_1493* : : *aphA-3*	This study
*ssx_1490*	Km^r^; Δ*ssa_1490* : : *aphA-3*	This study
*ssx_1489*	Km^r^; Δ*ssa_1489* : : *aphA-3*	This study
*ssx_2118*	Km^r^; Δ*tpk* : : *aphA-3*	This study
*ssx_2118*C	Cm^r^; *tpk+* : : *magellan2*	This study
*ssx_2120*	Km^r^; Δ*ssa_2120* : : *aphA-3*	This study
*ssx_2119*	Km^r^; Δ*ssa_2119* : : *aphA-3*	This study
*ssx_2117*	Km^r^; Δ*ssa_2117* : : *aphA-3*	This study
*ssx_2116*	Km^r^; Δ*ssa_2116* : : *aphA-3*	This study
***S. mutans***		
UA159	Wild-type, serotype *c*	ATCC 700610
*smx_42*	Cm^r^; Δ*smu.42* : : *magellan2*	This study
